# Global Progress and Challenges in Implementing New Medications for Treating Multidrug-Resistant Tuberculosis

**DOI:** 10.3201/eid2203.151430

**Published:** 2016-03

**Authors:** Jennifer Furin, Grania Brigden, Erica Lessem, Michael Rich, Laura Vaughan, Sharonann Lynch

**Affiliations:** Harvard Medical School, Boston, Massachusettes, USA (J. Furin); Médecins Sans Frontières Access Campaign, Geneva, Switzerland (G. Brigden, S. Lynch);; Treatment Action Group, New York, NY, USA (E. Lessem);; Brigham and Women’s Hospital, Boston (M. Rich);; Partners In Health, Boston (M. Rich, L. Vaughan)

**Keywords:** drug-resistant TB, bedaquiline, delamanid, global progress, Mycobacterium tuberculosis and other mycobacteria, bacteria, antimicrobial resistance

## Abstract

Two new drugs—bedaquiline and delamanid—have recently been approved by stringent regulatory authorities to treat multidrug-resistant tuberculosis (TB) and recommended by the World Health Organization for use under defined programmatic conditions. Introducing the medications in TB programs worldwide has not kept pace with the need for these drugs. In response, the DR-TB STAT (Drug-Resistant TB Scale-up Treatment Action Team) task force was formed in April 2015 to monitor progress and help overcome challenges. Information was collected from multiple sources and assessed monthly. Some progress has been made in introducing bedaquiline: as of October 2015, a total of 1,258 persons were on the medication under programmatic conditions. For delamanid, >100 patients, but few under programmatic conditions, have received the medication. Coordinated global action might help assist making these medications accessible for persons who need them most.

Multidrug-resistant (MDR) tuberculosis (TB) is a serious global public health problem (*1*), and estimates indicate that unless the management of MDR TB changes radically, it will be one of the main drivers of antimicrobial resistance, which could kill more persons than cancer by 2050 (*2*). Current therapy for MDR TB requires the use of multiple, toxic, and expensive drugs for 18–24 months and results in success in only about half of treated patients (*3*). The introduction of 2 new medications to treat MDR TB—bedaquiline (BDQ) and delamanid (DLM)—has renewed hope for improved individual outcomes and for stopping the ongoing transmission of MDR TB (*4*).

BDQ is a diarylquinoline agent (*5*) of Janssen Therapeutics (Titusville, NJ, USA) that, in December 2012, the US Food and Drug Administration conditionally recommended to treat MDR TB (*6*) after the results of a phase IIB randomized controlled trial showed faster time to culture conversion and higher rates of culture conversion at 6 months in patients who received the drug plus background regimen than in patients who received background regimen plus placebo (*7*). In June 2013, the World Health Organization (WHO) recommended BDQ for programmatic management of MDR TB when other alternatives are not adequate (*8*). DLM is a nitroimidazole agent (*9*) of Otsuka Pharmaceuticals (Princeton, NJ, USA) that, in April 2014, was granted conditional marketing authorization for treating MDR TB by the European Medicines Association (*10*) after a phase IIB trial with an open-label extension showed higher rates of culture conversion for persons receiving >2 months of DLM than for those receiving <2 months of the drug (*11*). In October 2014, WHO recommended use of DLM for treating MDR TB under specific conditions (*12*). WHO recommended both new drugs be used according to 5 criteria (Table 1). Although BDQ was generally recommended for patients with resistance or intolerance to the injectable agents, the fluoroquinolones, or both, DLM was additionally recommended for any MDR TB patient at high risk for poor treatment outcome.

**Table 1:** World Health Organization recommendations for the programmatic use of bedaquiline and delamanid to treat multidrug-resistant tuberculosis

Recommendation
1. The drug is used under carefully monitored conditions.
2. Patients to receive the drug are carefully selected.
3. The drug is used as part of a World Health Organization–recommended treatment regimen.
4. Patients to receive the drug sign an informed consent; for delamanid, the recommendation is only for “due process” for informed consent.
5. Adverse events, including active pharmacovigilance, are actively managed.

Despite the conditional approval of these 2 new medications by stringent regulatory authorities and the WHO recommendations, BDQ and DLM have yet to reach most patients worldwide who have indications for these drugs (*13*). The high death rates for MDR TB and the ongoing spread of the disease in communities (*14*)—especially those with high rates of HIV (*15*)—prompted >88 civil society groups to issue an open letter calling for more urgent action to make BDQ and DLM available globally (*16*). This letter outlined several key aspirational and time-bound targets to be achieved by the letter’s addressees (Table 2). In response, a discussion was held in Geneva as part of a planned Global Laboratory Initiative/Global Drug Resistance Initiative (GDI) meeting, and a task force of the GDI (DR-TB STAT [Drug-Resistant TB Scale-up Treatment Action Team]) was formed (*17*). The goal of DR-TB STAT is to monitor progress against set time-bound targets for introducing BDQ and DLM under programmatic conditions for treating MDR TB. We present findings of the task force on global progress and challenges encountered in new drug introduction.

**Table 2:** Summary points from the global call to action on the programmatic use of bedaquiline and delamanid to treat MDR TB

Point title	Explanation
1. Quickstart	Patients are started on routine regimens, which include DLM, by January 2016.
2. Optimal MDR TB treatment	Technical assistance is provided for 25 countries by 2016 and 52 countries by 2017 for drafting implementation plans; implementation plans are adopted by 25 countries by 2016 and 52 countries by 2018; and BDQ and DLM are routinely used by 20 countries by end of 2016 and 52 countries by end of 2019. Key repurposed drugs (especially linezolid and clofazimine) should be on the national Essential Medicines List, and countries and national TB program should be using these drugs.
3. Regulatory status	BDQ and DLM dossiers are submitted for registration in 25 countries by beginning of 2016 and 52 countries by 2017; and drugs are registered, or import waivers are in place, by 2016.
4. Pharmacovigilance	The consortium† supports a flexible approach for countries implementing BDQ (such as sentinel pharmacovigilance), proposes a set of standardized data for monitoring and reporting on adverse events, and works toward a supranational body to collect and analyze data.
5. Procurement	Forecasting of drugs is completed; procurement strategies are developed for 52 countries by 2018; and the turnaround time between ordering and drug delivery is reduced.

*BDQ, bedaquiline; DLM, delamanid; MDR TB, multidrug-resistant tuberculosis. †The DR-TB STAT (Drug-Resistant TB Scale-up Treatment Action Team) task force.

## Processes Used by DR-TB STAT

### Task Force

The DR-TB STAT task force comprises stakeholders working to introduce new medications for treating MDR TB (Table 3). DR-TB STAT officially became a task force of the GDI in July 2015. Although task force status was pending, DR-TB STAT held its initial meeting in April 2015, and since then has met monthly by conference call to discuss progress and challenges in new drug introduction generally and in regard to specific countries and programs.

**Table 3:** Alphabetical list of core organizations participating in DR-TB STAT

National TB program director	Implementing partner	Technical assistance provider	Donor	Advocacy group
Belarus	Global Drug Facility	Clinton Foundation	Global Fund for AIDS, TB, and Malaria	Global Coalition of TB Activists
India		Management Sciences for Health/Systems for Improved Access to Pharmaceutical and Services	UNITAID	Global TB Community Advisory Board
Russian Federation	KNCV Tuberculosis Foundation	Stop TB Partnership	US Agency for International Development	
South Africa	Médecins Sans Frontières	Society Working on Implementation to Fight TB Response Project		Moldovan Society Against Tuberculosis
Vietnam	Partners in Health	World Health Organization		RESULTS UK
				Treatment Action Campaign
				Treatment Action Group
*DR, drug-resistant; STAT, Scale-up Treatment Action Team; TB, tuberculosis.				

### Information Collected

Information was collected from multiple sources: national TB programs, country reports, WHO reports, information from Janssen Therapeutics and Otsuka Pharmaceuticals, a variety of donors (US Agency for International Development [USAID], UNITAID, and The Global Fund), order data from the Global Drug Facility, and reports from nongovernment organizations (e.g., Partners in Health, Médecins Sans Frontières, Treatment Action Group, and KNCV Tuberculosis Foundation). Data were collected and updated monthly by using a standard collection form that included the following variables: 1) the number of patients treated with each drug as part of compassionate use/expanded access programs, 2) the number of patients treated with each drug under programmatic conditions, 3) the number of countries using each drug under programmatic conditions, 4) the number of orders placed for each drug, 5) the number of countries in which each drug has been registered or in which registration is pending, and 6) the projected number of patients and countries that will be receiving each drug in 2016. Summary data were tabulated to describe the overall assessment of global progress that is described here. For this report, compassionate use was defined as use of the drug accessed from the company for a specific patient; expanded access was defined as use of the drug accessed from the company for a group of patients who meet certain criteria (*18*).

In addition to focusing on global progress, DR-TB STAT also helps identify and address barriers to new drug introduction both generally and in program-specific settings. Detailed field notes documented the challenges reported by participants in the DR-TB STAT discussions. These notes were then reviewed to identify barriers to introduction of BDQ and DLM under programmatic conditions by using a thematic analysis (*19*). For the thematic analysis, notes were reviewed by a trained qualitative scientist (J.F.) to identify patterns and recurring content until saturation was reached. The responses were then grouped into topic areas.

## Findings of the DR-TB STAT Task Force

### BDQ

**Figure 1:**
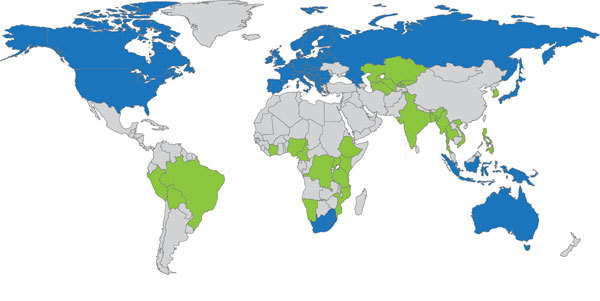
Global progress on programmatic use of bedaquiline (BDQ) to treat multidrug-resistant tuberculosis. Blue indicates countries using BDQ under program conditions. Green indicates countries awaiting arrival of BDQ to use it under program conditions. Gray indicates countries that have not reported using BDQ under program conditions.

As of October 1, 2015, >700 persons had received BDQ through compassionate use and expanded access. In addition, 1,258 persons had received BDQ under programmatic conditions in 9 countries plus the European Union (EU). Orders for BDQ had been placed through the GDF for 1,680 persons in 24 additional countries, and these programmatic treatments are expected to begin in the next 6 months (Table 4; Figure 1).

**Table 4:** World Health Organization–defined high-burden, low- and middle-income countries using or waiting for drug arrival to begin using bedaquiline under program conditions for treatment of multidrug-resistant tuberculosis

Currently using	Awaiting drug arrival
Armenia	Bangladesh
Belarus	Bolivia
Georgia	Brazil
Indonesia	Cameroon
Lesotho	Cote d’Ivoire
Papua New Guinea	North Korea
Russia	Democratic Republic of the Congo
South Africa	Ethiopia
Swaziland	India
	Kazakhstan
	Kenya
	Kyrgyzstan
	Mozambique
	Myanmar
	Namibia
	Nigeria
	Peru
	Philippines
	South Korea
	Thailand
	Turkmenistan
	United Republic of Tanzania
	Uzbekistan
	Vietnam

In terms of registration, BDQ has been registered in the EU (28 countries) and 13 additional countries. Registration is pending in 13 other countries. Based on countries’ reported plans and the plans of other implementing groups, BDQ possibly could be given to a cumulative total of >7,000 persons under programmatic conditions by the end of 2016. Turnaround time from placement of order to drug delivery was 3–6 months

### DLM

**Figure 2:**
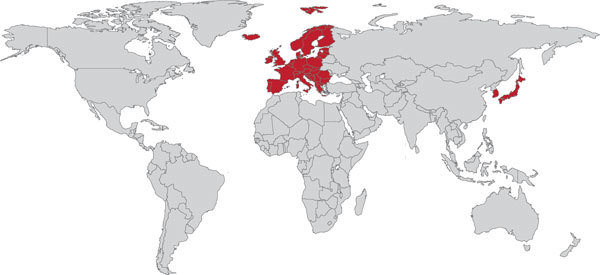
Global progress on the programmatic use of delamanid (DLM) to treat multidrug-resistant tuberculosis. Red shading indicates countries using DLM under program conditions. Gray indicates countries that have not reported using DLM under program conditions.

Despite repeated requests, Otsuka Pharmaceuticals did not provide specific information about DLM access, citing this information as proprietary. Therefore, data on the use of DLM were compiled from other sources. According to the company, as of October 1, 2015, >100 patients had received DLM through compassionate use. DLM orders cannot be placed through the GDF, and Otsuka Pharmaceuticals did not provide information about pending drug orders (Figure 2).

DLM has been registered in the EU, Japan, and South Korea. No information was found about pending registration in any additional countries. According to countries’ reported plans and the plans of other implementing groups, DLM could potentially be given to >550 persons under program conditions by the end of 2016. No information about turnaround time for drug orders could be obtained.

## Challenges in Using New Drugs Under Programmatic Conditions

Data collected on barriers to use of new drugs that were discussed during DR-TB STAT meetings revealed a variety of reported challenges to using new drugs under programmatic conditions. In general, the problems reported fell into 10 areas: 1) lack of awareness of drug availability and procurement process; 2) limited availability of adequate technical expertise; 3) confusion around WHO “requirements,” most notably pharmacovigilance; 4) limited availability of quality clinical trials data supporting the use of new drugs under programmatic conditions; 5) challenges in sharing rapidly changing information about new drugs with key stakeholders and incorporating such information into national guidelines; 6) concerns that the process of new drug introduction is “too complicated” under programmatic conditions; 7) prolonged turnaround time for drug procurement; 8) difficulties in import and customs clearance; 9) limited access to companion MDR TB medications, especially linezolid and clofazimine; and 10) lack of high-level national government support.

Many of the goals toward achieving the targets in the global call to action have been met for BDQ. However, the goals set for DLM are unlikely to be met (Table 5). 

**Table 5:** Progress in achieving targets set in global call to action for the programmatic use of bedaquiline and delamanid to treat multidrug-resistant tuberculosis

Target	Status
500 Patients on BDQ by July 2015	1,258 patients by October 1, 2015
500 Patients on DLM by Jan 2016	>100 patients by October 1, 2015
TA given to 25 countries by 2016	Ongoing, already provided in 21 countries
BDQ and DLM routinely used by 20 countries by end of 2016	BDQ: 9 countries plus European Union by October 1, 2015; additional 20 by the mid-2016
	DLM: 2 countries plus European Union by October 1, 2015; plans for other countries expected to be discussed in December 2015
BDQ and DLM dossiers submitted for registration in 25 countries by beginning of 2016	BDQ: 26 countries by October 1, 2015
	DLM: 3 countries by October 1 2015

*BDQ, bedaquiline; DLM, delamanid.

## Discussion

Although MDR TB is treatable and curable, managing it under program conditions has long been characterized by a host of problems. These include challenges in the detection of resistance, especially to second-line drugs; delays in prompt initiation of appropriate therapy; and insufficient mechanisms for ongoing monitoring for toxicity and continued engagement of patients throughout the 18–24-month course of treatment. Furthermore, challenges exist to staff training and retention, information management, and infection control. Access to new drugs will not erase these barriers, but access does offer hope for improving individual patient outcomes and decreasing the transmission of MDR TB. Uptake of BDQ and DLM under programmatic conditions, however, has not kept pace with need. According to WHO recommendations for the use of these medications, 25%–50% of MDR TB patients meet criteria to receive these drugs because of their high-level resistance, intolerance to drugs currently used to treat MDR TB, or risks for poor outcomes (*20*). The occurrence of ≈500,000 new cases of MDR TB each year means that 125,000–250,000 new patients would benefit from the use of either of these drugs each year, a number that does not include the large number of prevalent MDR TB patients who might benefit from new treatment options. More conservative estimates of the need for new drugs using the actual numbers of MDR TB patients placed on treatment each year show that 24,250–48,500 of the 96,000 persons started on treatment (*21*) would benefit from access. Even using these less ambitious numbers, it is clear that more work needs to be done to improve access to BDQ and DLM.

The findings of DR-TB STAT show that more persons with MDR TB who have an indication for new drugs have access to BDQ than to DLM. Wider access to BDQ is occurring even though the WHO recommendations for DLM use are broader and despite the association of BDQ with a higher death rate in its phase IIB trial. For several reasons, access might be greater to BDQ than to DLM. BDQ was conditionally approved by a stringent regulatory authority and recommended by WHO >1 year before DLM was approved (*22*); over time, access to both drugs might become more comparable. However, several other possible explanations exist. First, the compassionate use/expanded access program for BDQ started nearly 3 years earlier and was more extensive and systematic than that for DLM. As a result, multiple countries and clinical providers have had experience with BDQ and thus might be more comfortable using it on a broader scale (*23*). Second, BDQ is registered in more countries than DLM; thus programmatic use might be more straightforward. Third, BDQ also is available through the GDF, whereas DLM can be obtained directly only from the company, although the company’s website contains no information about how the drug can be purchased (*24*). Finally, in March 2015, USAID began implementing a BDQ donation program in which 30,000 treatment courses are provided free of charge for 4 years, along with the technical assistance that countries might need to implement the drug (*25*). Although Otsuka Pharmaceuticals announced a “20 by 2020” access initiative—meaning 20% of MDR TB patients worldwide would receive DLM by 2020 (*26*)—the company has not provided information about how this program will work.

Stakeholders participating in the DR-TB STAT meetings discussed multiple barriers and challenges to new drug implementation that also appear to be slowing new drug introduction. Several of these seem to be misconceptions about how to access or use the drugs, including confusion about processes for procurement and import, the specific WHO recommendations—especially around pharmacovigilance—for the use of new drugs, and beliefs that the drugs cannot be used under programmatic conditions. Indeed, all stakeholders participating in DR-TB STAT listed lack of access to technical assistance from experienced providers, including provision of rapidly changing information about new drug introduction, as a major challenge. These challenges could be addressed by providing global training for programs and their implementing partners. One approach to filling this gap would be using innovative training tools, including Web-based training. Plans for offering such training globally are now being coordinated by the DR-TB STAT participants with the hope that much of the confusion about how to optimally use BDQ and DLM under programmatic conditions can be eased.

Other challenges in new drug implementation stem from lack of access to BDQ and DLM and the other drugs needed to form complete treatment regimens. Increased registration and additional experience importing the medications also should help the drugs clear customs more rapidly. The inclusion of both drugs, along with linezolid, on the WHO Model Essential Drugs List in April 2015 might help boost country confidence and registration for their use in additional countries.

Finally, improved access to companion drugs used with BDQ and DLM, especially linezolid and clofazimine, is urgently needed. Until sufficient evidence enables BDQ and DLM to be combined, linezolid and clofazimine (and other “group 5” drugs) are used to ensure that the new drugs are not added singly to a failing regimen. These companion drugs are expensive—in the case of linezolid, more so than BDQ—and are not registered with an MDR TB indication in most of the countries using BDQ and DLM. Similar attention needs to be given to these drugs, and countries need to be supported in accessing them. DR-TB STAT is likely to begin formally monitoring and assisting countries with access to these medications in 2016.

Access to new drugs alone will not solve the ongoing crisis of MDR TB, and much more comprehensive action is needed to improve care for persons with this disease on a global level (*27*). These medications, however, are one of the few current hopes for more effective and less toxic therapy for MDR TB–affected persons worldwide (*28*). Coordinated efforts can help ensure timely and equitable access to BDQ and DLM for persons who need them most.
